# Predicted sub-populations in a marine shrimp proteome as revealed by combined EST and cDNA data from multiple *Penaeus *species

**DOI:** 10.1186/1756-0500-3-295

**Published:** 2010-11-11

**Authors:** Pimlapas Leekitcharoenphon, Udon Taweemuang, Prasit Palittapongarnpim, Rattanawadee Kotewong, Thararat Supasiri, Burachai Sonthayanon

**Affiliations:** 1 Center of Excellence for Shrimp Molecular Biology and Biotechnology (CENTEX Shrimp), Faculty of Science, Mahidol University, Rama VI Road, Bangkok 10400, Thailand; 2 National Center for Genetic Engineering and Biotechnology (BIOTEC), National Science and Technology Development Agency (NSTDA), Ministry of Science and Technology, Thailand Science Park, 113 Paholyothin Road, Tambon Khlong 1, Amphoe Khlong Luang, Pathum Thani 12120, Thailand; 3 Department of Microbiology, Faculty of Science, Mahidol University, Rama VI Road, Bangkok 10400, Thailand; 4 Department of Chemistry, Srinakharinwirot University, Sukhumvit 23 Rd., Bangkok 10110, Thailand

## Abstract

**Background:**

Many species of marine shrimp in the Family Penaeidae, viz. *Penaeus (Litopenaeus) vannamei, Penaeus monodon, Penaeus (Fenneropenaeus) chinensis*, and *Penaeus (Marsupenaeus) japonicus*, are animals of economic importance in the aquaculture industry. Yet information about their DNA and protein sequences is lacking. In order to predict their collective proteome, we combined over 270,000 available EST and cDNA sequences from the 4 shrimp species with all protein sequences of *Drosophila melanogaster *and *Caenorhabditis elegans*. EST data from 4 other crustaceans, the crab *Carcinus maenas*, the lobster *Homarus americanus *(Decapoda), the water flea *Daphnia pulex*, and the brine shrimp *Artemia franciscana *were also used.

**Findings:**

Similarity searches from EST collections of the 4 shrimp species matched 64% of the protein sequences of the fruit fly, but only 45% of nematode proteins, indicating that the shrimp proteome content is more similar to that of an insect than a nematode. Combined results with 4 additional non-shrimp crustaceans increased matching to 78% of fruit fly and 56% of nematode proteins, suggesting that present shrimp EST collections still lack sequences for many conserved crustacean proteins. Analysis of matching data revealed the presence of 4 EST groups from shrimp, namely sequences for proteins that are both fruit fly-like and nematode-like, fruit fly-like only, nematode-like only, and non-matching. Gene ontology profiles of proteins for the 3 matching EST groups were analyzed. For non-matching ESTs, a small fraction matched protein sequences from other species in the UniProt database, including other crustacean-specific proteins.

**Conclusions:**

Shrimp ESTs indicated that the shrimp proteome is comprised of sub-populations of proteins similar to those common to both insect and nematode models, those present specifically in either model, or neither. Combining small EST collections from related species to compensate for their small size allowed prediction of conserved expressed protein components encoded by their uncharacterized genomes. The organized data should be useful for transferring annotation data from model species into shrimp data and for further studies on shrimp proteins with particular functions or groups.

## Findings

### Background

Marine shrimp (order Decapoda, family Penaeidae) are crustaceans of high economic importance, notably the Pacific whiteleg shrimp *Penaeus (Litopenaeus) *vannamei and the giant tiger shrimp *Penaeus (Penaeus) monodon*, that are prominent species in the shrimp aquaculture industry of several countries in Asia Pacific and the Americas [1]. Other species raised include Chinese shrimp *Penaeus (Fenneropenaeus) *chinensis and Kuruma shrimp *Penaeus (Marsupenaeus) *japonicus [2]. Despite the multibillion dollar size of the industry for each country, molecular studies at the nucleotide and protein sequence levels of marine Penaeid shrimp and other Decapod crustaceans are still considered inadequate for investigating numerous farm level problems related to viral and bacterial pathogenesis. Relatively few DNA and protein sequence entries from true marine shrimp and decapods are present in sequence databases such as GenBank when compared to those from insects and other groups of animals [3]. As of today, no complete decapod crustacean genome sequence has been published, although the genome sequencing project of a copepod crustacean, *Daphnia pulex*, is complete [4-6]. The majority of crustacean sequences available in primary sequence databases are in small collections as single-pass partial cDNA sequences known as expressed sequence tags or ESTs accessible via GenBank's dbEST database as well as via species-specific EST databases [4,7-9]. However, a number of insect genome sequences from the same Arthropoda phylum have been published and released such as those of *Drosophila melanogaster *[10], *Anopheles gambiae *[11], *Aedes aegypti *[12], and *Tribolium castaneum *[13]. Genomes of lower eukaryotes that have been characterized include those of *Caenorhabditis elegans *[14], *Caenorhabditis briggsae *[15], and *Strongylocentrotus purpuratus *[16]. Among these, the best studied invertebrate models are *D. melanogaster *and *C. elegans*.

Full-length and partial cDNA sequences have provided useful snapshots of the protein-coding regions of genomes [17]. However, the number of crustacean full length or partial cDNA sequences in GenBank are in the lower hundred range. Work on shrimp cDNA libraries to generate ESTs have led to characterization of a number of protein coding sequences from isolated full-length cDNA clones [18-22]. Despite the lack of a sufficient number of full length cDNAs, there are small sets of released EST data from a number of species available from public databases. Of interest to the aquaculture industry are studies conducted on *P. monodon, P. vannamei, P. chinensis*, and *P. japonicus*, the former two having sizable collections of EST sequences [8,9,23,24]. Publications about ESTs often mention a selection of their sequences that have similarity to known entries from other species in databases. However, the coverage of genome-wide proteomes are often not reported. Yet the issue is of interest for researchers and research managers since it could signify how much more effort should be given to generate additional diverse EST + cDNA data to ensure that they cover all the proteins that might be useful for biotechnology applications.

Genome-wide proteome content provides useful information for delimitation of biochemical functions to be expected in cells from a given living species. To predict the scope of a proteome, a comprehensive collection of cDNAs is generally required. Unfortunately, the existing cDNA and EST collections from each shrimp species are small, so the scope of the shrimp proteome has not been previously addressed. For the two prominent aquaculture species, *P. vannamei *and *P. monodon*, there are only around 160,000 and 97,000 ESTs, respectively. For 2 other lesser cultured species, *P. chinensis *and *P. japonicus*, there are around 10,000 and 3,000 ESTs, respectively. To study the scope of the expressed shrimp proteome, we decided to overcome the shortcoming of small EST collections by combining data from the species with sizable collections and to analyze them as a representative model for the Penaeid shrimp group. By comparing them with whole-genome protein sequences from the two well-studied models in the phyla Arthropoda and Nematoda using the BLAST program [25], we found evidence that the collective shrimp or crustacean proteome is more similar to the proteome of an insect than a nematode. Almost ten thousand proteins of *D. melanogaster *and *C. elegans *were predicted to have similar proteins in shrimp and Decapods. We also estimated the extent of shortcoming in shrimp EST collections. More importantly, we predicted that the shrimp proteome could be subdivided into groups, one that has protein sequences similar to those found in both the insect and nematode, one that has protein sequences similar to only the insect proteins, one that has protein sequences similar to only the nematode proteins, and one that has protein sequences similar to neither of these models. This unexpected new finding is noteworthy for people working with crustacean genes or genomes. Lastly, features of the predicted protein coding sequences in matching EST groups were analyzed for their functional profiles by GO analysis [26-28].

## Methods

EST data were obtained mainly from GenBank dbEST (downloaded on August 10, 2009) [3,7]. For *P. monodon*, data sets from *Penaeus monodon *EST project database were also added [8,9]. Available cDNA sequences from GenBank for *P. monodon *(415 sequences) and *P. vannamei *(339 sequences, accessed October 29, 2009) were added to the EST data for each species. The shrimp species analyzed included the giant tiger shrimp *P. monodon *(97,805 ESTs + 415 cDNAs), the Pacific whiteleg shrimp *P. vannamei *(160,381 ESTs + 339 cDNAs), fleshy shrimp *P. chinensis *(10,446 ESTs), and Kuruma shrimp *P. japonicus *(3,152 ESTs), totaling 272,538 ESTs for the 4 shrimp species. Other Penaeid shrimp species having small numbers of EST and cDNA were not included in this study. Other Decapod sequences from dbEST were from the Atlantic lobster *Homarus americanus *(29,558 ESTs), and the littoral crab *Carcinus maenas *(15,558 ESTs). Total ESTs for all 6 Decapoda species were 317,654 entries. Non-Decapod primitive crustaceans included were brine shrimp *Artemia franciscana *(37,487 ESTs), and the water flea *Daphnia pulex *(165,917 ESTs). Protein coding sequences from the fruit fly *D. melanogaster *(20,815 sequences, version 5.4) and the nematode *C. elegans *(27,258 sequences, data version 190) were obtained from Ensemble database [29]. Predicted protein sequence set for *D. pulex *was obtained from wFleaBase [4]. Protein data of all living species were obtained from UniProt (accessed October 30, 2009), comprising 509,019 protein sequences [30].

BLASTX and TBLASTN programs (version 2.2.18) were performed in a Linux computational cluster at the National Center for Genetic Engineering and Biotechnology (BIOTEC), Thailand, with a cut-off E value set at 10^-4^. Queries between DNA sequences from each crustacean species and protein sequences from each model species were computed separately. Outputs from BLAST analyses were parsed by Perl scripts using BioPerl code modules [31]. Grouping of EST data was performed using a Python script to extract just best-hit entries for each query from BLASTX and TBLASTN results. GO mappings were conducted using a perl script to traverse through graph structure of ontology data using a publicly available go-perl module and existing full GO annotation data from FlyBase and WormBase [27,28].

## Results and Discussion

**Figure 1:**
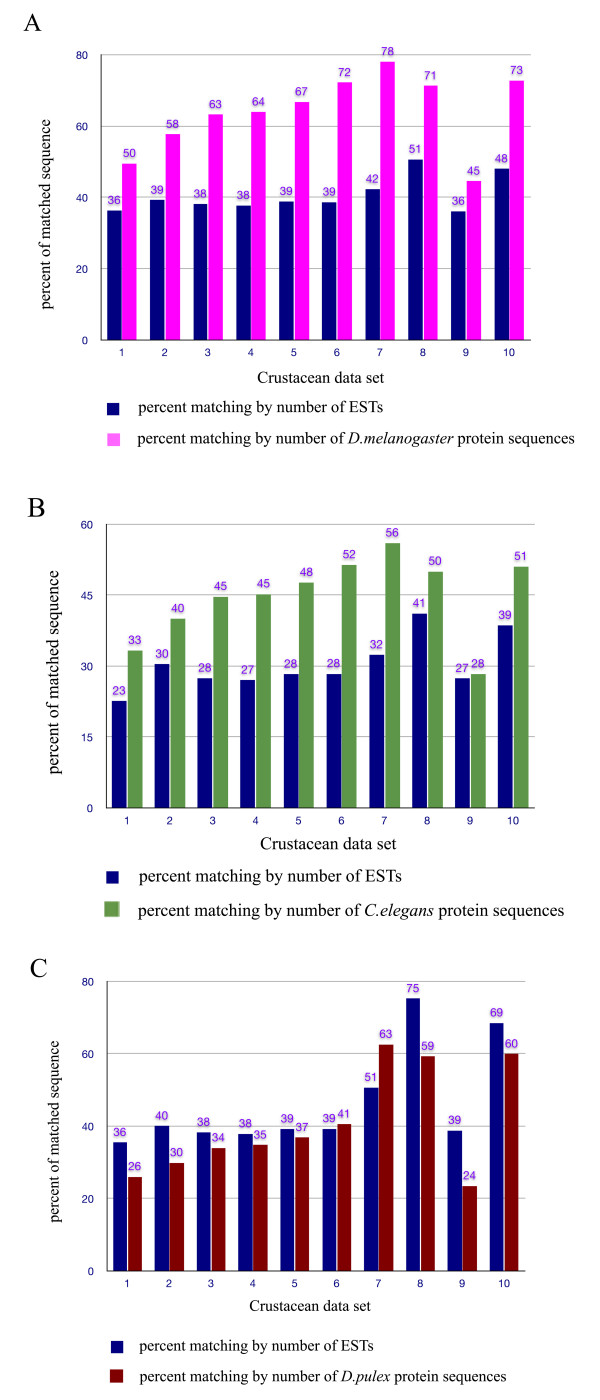
**Matching between shrimp and crustacean ESTs and proteins from model species**. Matching percentages obtained from BLASTX and TBLASTN results are shown relative to the total number of crustacean EST sequences in each set (dark blue bars) or to the total number of protein sequences in a model species (pink bars for *D. melanogaster*, green bars for *C. elegans*, dark red bars for *Daphnia pulex*). Data sets were either for single crustacean species or multiple species as follows:- set 1 : *P. monodon *(total = 98,220 ESTs + cDNAs). set 2 : *P. vannamei *(total = 160,720 ESTs + cDNAs). set 3 : *P. monodon *and P. vannamei (total = 258,940 ESTs + cDNAs). set 4 : *P. monodon, P. vannamei, P. chinensis *and *P. japonicus *(total = 272,538 sequences). set 5 : 4 true shrimp species + 1 decapod species, *P. monodon, P. vannamei, P. chinensis, P. japonicus *and *C. maenas *(total = 288,096 sequences). set 6 : 4 true shrimp species + 2 decapod species, *P. monodon, P. vannamei, P. chinensis, P. japonicus, C. maenas *and *H. americanus *(total = 317,654 sequences). set 7 : 4 true shrimp species + 4 other crustacean species, *P. monodon, P. vannamei, P. chinensis, P. japonicus, C. maenas, H. americanus, A. franciscana *and *D. pulex *(total = 521,058 sequences). set 8 : *D. pulex *(total = 165,917 ESTs). set 9 : *A. franciscana *(total = 37,487 ESTs). set 10 : *D. pulex *and *A. franciscana *(total = 203,404 ESTs). **(A) Matching when comparing with 20,815 protein sequences of *D. melanogaster***. **(B) Matching when comparing with 27,258 protein sequences of *C. elegans***. **(C) Matching when comparing with 37,466 protein sequences of *D. pulex***

The scope of the shrimp expressed proteome was determined by running BLASTX and TBLASTN (E < 10^-4^) between the DNA sequence collection from each shrimp species and the protein sequences from each model species, *D. melanogaster*, and *C. elegans*. Matching results for each shrimp species returned by the two programs were combined. Comparison of those with proteins from a crustacean model, *Daphnia pulex*, was also conducted as a control. Figure 1 shows percentages of matchings between crustacean EST collections and protein sequences from the model species. For EST + cDNA from both of the major aquaculture species, *P. monodon *or *P. vannamei*, 36-39% of ESTs gave significant similarity to reference protein sequences, constituting 50-58% of all the proteins in the fruit fly (Figure 1A, data sets 1-2). When shrimp ESTs were compared to proteins from the nematode, 23-30% of the ESTs from either *P. monodon *or *P. vannamei *gave significant matchings with only 33-40% of nematode proteins (Figure 1B, data sets 1-2). When data from the 2 shrimp species were combined, the percentage of protein similarity with the fruit fly and nematode proteins rose significantly to 63% and 45%, respectively (Figure 1A, B, data set 3, pink and green bars). When smaller sets of EST data from the 2 other lesser farmed shrimp species, *P. japonicus *and *P. chinensis *were added to create a 4 shrimp species set, the percentage of protein matchings with those of fruit fly and nematode rose slightly to 64% and 45%, respectively (Figure 1A, B, data set 4, pink and green bars). As farmed Penaeid shrimp are decapods, addition of EST data from 2 more decapod species (*Homarus americanus *and *Carcinus maenas*) to constitute a 6-species Decapod data set, the percentage of similarity with fruit fly and the nematode proteins rose to 72% and 52%, respectively (Figure 1A, B, data set 6, pink and green bars). If EST data from 2 small crustacean species, *Daphnia pulex *and brine shrimp *Artemia franciscana *were also added to generate an 8 species crustacean EST data set, the percentages of similarity matchings increased to 78% and 56% of fruit fly and nematode proteins, respectively (Figure 1A, B, data set 7, pink and green bars). These results showed a trend that the matching sequences of shrimp and other crustaceans are more similar to proteome sequences of the fruit fly than nematode. This result was not surprising, given that crustaceans are classified in the same phylum Arthropoda as insects. Nevertheless, this result is special since we used a trend of genome-wide expressed sequence data, not just few markers, to support the notion of more similarity between crustaceans and insects than crustaceans and nematodes. The result also suggested that the majority of the fruit fly proteins (64-78%) might have similar proteins in shrimp and crustaceans (Figure 1A, data sets 4-7, pink bars). This implied that a protein set from the fruit fly, if selected for those with matching ESTs from shrimp and crustaceans, might be used as a Systems Biology data model for shrimp and crustaceans.

We then proceeded to determine if existing shrimp EST collections had covered all the expressed proteome or not. If ESTs from only 4 shrimp were grouped, they gave a 64% match with fruit fly protein sequences. However, when EST data from all 8 crustaceans were grouped, they gave 78% matching. The 14% difference suggested that the existing EST data from the 4 *Penaeus *species still lack sequences of around 14% of the conserved pan-crustacean proteins. If the percent of fruit fly protein hits by ESTs from 6 species of decapods (72%) is considered, the difference is 8% (Figure 1A, data sets 4,6). Again, this suggested that the current set of EST data from economic shrimp lacks representation from around 8% of conserved decapod proteins, not taking into account possible sequence divergences among thousands of shrimp and decapod species. As a control for comparison, the EST set from the model crustacean, *Daphnia pulex*, which has the largest EST collection among crustaceans in dbEST, gave 71% similarity to fruit fly proteins (Figure 1A, data set 8, pink bar). When EST data from *Artemia *were added to the *Daphnia *data set, similarity rose slightly to 73% (Figure 1A, data set 10, pink bar), still lower than the 78% from the result of 8 combined crustacean species (data set 7, pink bar), yet about the same value of 72% obtained using the combined data from 6 decapods. So, 8% lack of representation of conserved proteins in the shrimp EST and cDNA collections seems to be a good approximation.

We were also interested in comparing shrimp ESTs with sequences of predicted proteins from the crustacean model, *D. pulex *from wFleaBase (Figure 1C). The percent matching by number of EST from each shrimp species or in combination with decapod ESTs were similar to the respective matching percentages with fruit fly proteins (Figure 1C, dark blue bars for data sets 1-6). However, the matching percentages by the number of predicted proteins in *D. pulex *were lower (Figure 1C, red bars, data sets 1-6) than those in *D. melanogaster*, possibly due to the much higher number of predicted protein sequences in this species (37,466 sequences) compared to fruit fly (20,815 sequences) and worm proteins (27,258 sequences). The higher percentage of matchings for nucleotides from either *D. pulex *or *A. franciscana*, or from both, to predicted protein sequences from *D. pulex *(as observed in lanes 8,9,10) may be due to the presence of more redundant ESTs from the two collections. EST Data combined from 6 decapod species gave only 41% matching by number of *Daphnia proteins*. Interestingly, ESTs from *D. pulex *had only 75% matching by ESTs, and 59% matching by predicted *Daphnia *proteins (Figure 1C, data set 8, blue and red bars). This suggested that there was a practical upper limit of matching between an EST data set and genome-wide predicted protein sequences, even for data from the same species. Therefore, the matching of combined ESTs from 8 crustacean species to 78% (16,268 proteins) of fruit fly proteins should be considered very high. The analysis showed that this approach of using combined EST + cDNA data from multiple related species to compensate for the lack of cloned low-abundant transcripts, especially from species with small EST collections in sequence databases, provided useful global information on the evolutionarily-conserved proteome content of a group of related species.

**Figure 2:**
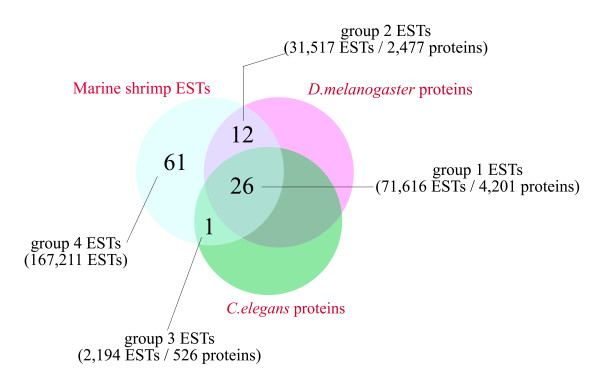
**Sub-populations of shrimp ESTs matching or not matching to protein sequences from the 2 model species**. Venn's diagram depicts grouping of ESTs from 4 *Penaeus *species which either matched as best-hits or did not match to protein sequences from *D. melanogaster *and/or *C. elegans*. (Intersecting areas representing EST percentages were not drawn to scale).

With the current focus on shrimp species of economic importance, we further analyzed ESTs from only the 4 Penaeid shrimp species based on the presence or absence of best hit proteins with model species in order to predict the scope of the proteome of shrimp. As shown in the Venn's diagram of Figure 2, 61% of the combined ESTs from 4 shrimp species did not match fruit fly or nematode protein sequences. Around 38% (12% + 26%) of the shrimp ESTs had best similarity matchings with fruit fly protein sequences and just 27% (26% + 1%) with nematode protein sequences. The matched EST population from shrimp could thus be divided into 3 groups, namely group 1 ESTs (26%) with matching proteins present in both the fruit fly and nematode [Additional file 1]; group 2 ESTs (12%), present in the fruit fly but not the nematode [Additional file 2]; and group 3 ESTs (1%) present in the nematode but not the fruit fly [Additional file 3]. However, these percentage values shown by EST numbers for each group need to be interpreted with care since EST records in databases are known to be redundant. This is because the EST records come from sequencing of randomly picked clones from various types of cDNA libraries. Assuming that their discovery frequency depends approximately on the relative abundance of each mRNA species in cell sources, one would expect to have more redundant ESTs from highly expressed transcripts than from lowly or conditionally expressed transcripts. Therefore, more useful information would be the corresponding number of best matched proteins predicted from the model species that corresponded to each EST group. The best-matched fruit fly-like and nematode-like proteins for group 1 ESTs of shrimp were equivalent to 4,201 fruit fly proteins, equivalent to around 20.2% of fruit fly protein sequences, and the best matched fruit fly-like only proteins for group 2 ESTs were equivalent to 2,477 fruit fly proteins (11.9% of fruit fly protein sequences). The best matched nematode-like only proteins for group 3 ESTs of shrimp were equivalent to 526 (1.9%) of nematode proteins. The total number of proteins from the 2 model species matched by the currently-available shrimp ESTs were equivalent to 7,204 (fruit fly + worm) proteins. If data from 2 additional decapod species were added, the 3 matching groups increased to 9,621 proteins (data not shown). This also implied that, compared to EST data from 2 Decapoda species, the current collection of shrimp ESTs still does not account for around 2,400 conserved Decapod proteins. With newly improved sequencing technology, the limited number of ESTs for *P. monodon *and *P. vannamei *will hopefully be resolved in the near future. In spite of the current shortcomings of shrimp ESTs, the results suggested that protein sequences from *D. melanogaster *and *C. elegans *which matched to the 3 EST groups are both needed to build a proteome model in shrimp and decapods. We also envisage that the 3 groups of shrimp and decapod ESTs that matched *Drosophila *and *Caenorhabditis *proteins will serve as a useful set of sequences for further research in transcriptomic or proteomic studies, or for selection of DNA markers toward construction of a genetic linkage map.

**Figure 3:**
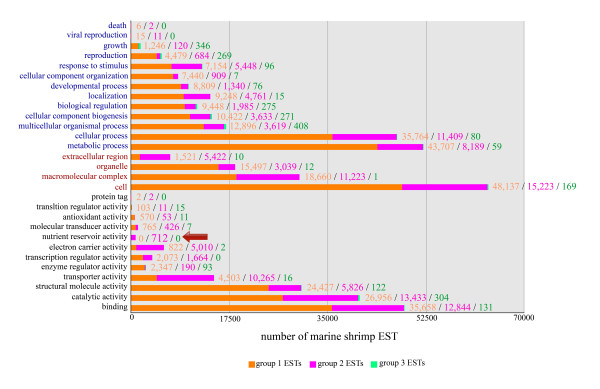
**Distribution of Gene Ontology categories of ESTs from shrimp**. Frequency of GO classes for EST groups 1, 2, 3 (orange bars, pink bars, green bars) from 4 *Penaeus *species are shown in 3 organized categories (from top to bottom): biological processes (blue labels), cellular components (red labels), and molecular function (black labels). Within each category, the first level ontology classes are shown by (increasing) frequency of EST counts. Classification of GO are based on those assigned to best-matched fruit fly or nematode proteins by FlyBase or WormBase. The red arrow points to a class found exclusively in group 2 ESTs.

To determine functional profiles of proteins in the predicted shrimp proteome, we decided to use only the matched ESTs from the 4 Penaeid shrimp species in the profile analysis. The ESTs were matched to the best-hit proteins and existing annotation information for the proteins from either *D. melanogaster *or *C. elegans *to create the first level Gene Ontology (GO) classes. The 3 groups of shrimp ESTs were then individually analyzed for their gene ontology functional profiles (Figure 3).

The GO distribution profile of the group 1 ESTs of Penaeid shrimp (matching both fruit fly and nematode proteins), constituting 26% (71,616 ESTs) of the EST data and best-matched with 4,201 fruit fly proteins gave the most of annotated functions (Figure 3, orange bars) [Additional file 4]. The group 2 ESTs (fruit fly protein-like only) showed a lesser number of annotated functions, yet it uniquely harbored a notable function group called nutrient reservoir activity (Figure 3, pink bar, red arrow) [Additional file 5]. Proteins with house-keeping functions, such as ribosomal proteins, were found in these 2 groups. The group 3 ESTs of shrimp (matching only nematode proteins, Figure 3, green bar) showed the least number of annotated functions [Additional file 6]. Although only 1% of EST data were in this group, this amounted to 526 nematode proteins, which is not a negligible number. We are tempted to speculate that group 3 ESTs might correspond to ancient proteins that have been lost in insects during the course of evolution, but have been retained in shrimp. From the protein names in the 3 EST groups, we could identify over a thousand fruit fly proteins that lacked matching ESTs in shrimp or decapods, including complexin, hephaestus, ewg, dachshund and dynamin. It is too early to tell whether shrimp actually lack these proteins, or whether their cDNAs simply have not been isolated and characterized so far.

**Figure 4:**
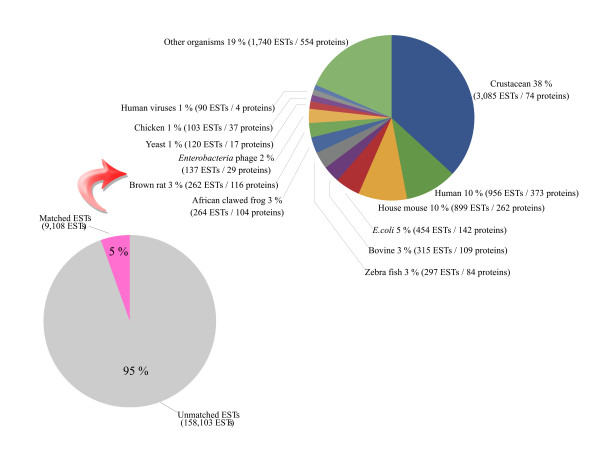
**Distribution of unmatched shrimp ESTs, a portion of which matched protein sequences from other species**. Around 61% of shrimp ESTs that did not match to fruit fly or nematode proteins were compared to protein sequences from UniProt using BLASTX and TBLASTN. Only 5% matched protein sequences from other species. The data sector designated as crustacean encompassed several species.

In order to determine whether the 61% unmatched EST population combined from 4 shrimp species had any sequence similarity to proteins from other species [Additional file 7], they were compared with all protein sequences in the UniProt databases using BLASTX and TBLASTN. Only 5% (9,108 ESTs) matched known proteins from other species (Figure 4) [Additional file 8]. The majority of newly-matched sequences were known crustacean protein sequences (38%, multiple species). Matched proteins from other species included those from humans (10%), the house mouse (10%), the cow (3%), zebra fish (3%), the African clawed frog (3%), the brown rat (3%), bacteriophages (2%), yeasts (1%), human viruses (1%), and other species (19%). Of the 5% of ESTs matching *E. coli *sequences, some might be contaminating sequences from cloning vectors. The predominant portion of the set of unmatched shrimp ESTs might still include some less-conserved proteins that would be useful for further studies.

## Conclusion

Our analysis shows the benefit of combining ESTs from related shrimp species to compensate for the small collection size of individual species and allow for the prediction of a conserved shrimp proteome model. Comparing EST sequences with whole-genome proteomes in model species allowed an assessment of the degree of coverage in existing EST collections for shrimp. Grouping of matching results provided evidence that predicted protein sets from shrimp and other crustaceans are more similar to those of an insect than a nematode. Furthermore, it revealed sub-populations of proteins similar to those common to both insect and nematode models, those present specifically in either model, or those present in neither. Slightly different profiles among the 3 matching EST groups were also observed from mapped GO functions. Our results suggest that conserved proteins in the 3 EST groups would be useful for transferring of annotation data from both model species to shrimp, for facilitating interpretations in microarray studies, for selection of cDNA clones to be used as genetic markers, and for further studies in shrimp proteins with particular functions or in particular groups.

## Competing interests

The authors declare that they have no competing interests.

## Authors' contributions

BS, PP, and TS planned the project and interpreted the results. PL, UT, RK conducted the experiments. BS drafted the manuscript. All authors read and approved the final manuscript.

## Supplementary Material

Additional file 1 **List of shrimps' group 1 ESTs which matched to protein sequences from both *D. melanogaster *and *C. elegans *(showing target proteins)**. This file lists 71,616 EST ID numbers (dbEST or other database-specific ID) and their matched protein ID with protein names (where available). Click here for file

Additional file 2 **List of shrimps' group 2 ESTs which matched to protein sequences from *D. melanogaster *only and their best-hit fruit fly protein ID**. This file lists 31,517 EST ID numbers (dbEST or other database-specific ID) and their best hit proteins with protein names (where available). Click here for file

Additional file 3 **List of shrimps' group 3 ESTs which matched to *C. elegans *protein sequences (showing hit proteins)**. This file lists 2,194 EST ID numbers (dbEST or other database-specific ID) and their matched WormBase ID and names of *C. elegans *proteins (where available). Click here for file

Additional file 4 **List of shrimps' group 1 ESTs which matched to protein sequences from both *D. melanogaster *and *C. elegans *and GO function**. This file lists 71,616 EST ID numbers (dbEST or other database-specific ID) and their first level GO function based on assignments to matched *D. melanogaster *proteins. Click here for file

Additional file 5 **List of shrimp-only ESTs which matched to *D. melanogaster *protein sequences and GO function (group 2 ESTs)**. This file lists 31,517 EST ID numbers (dbEST or other database-specific ID) and their first level GO function based on matched *D. melanogaster *proteins. Click here for file

Additional file 6 **List of shrimps' group 3 ESTs which matched to proteins from *C. elegans *and GO function**. This file lists 2,194 EST ID numbers (dbEST or other database-specific ID) and their first level GO function based on matched *C. elegans *proteins. Click here for file

Additional file 7 **List of non-matched shrimp ESTs which did not matched to any *D. melanogaster *or *C. elegans *proteins**. This file lists 167,211 EST ID not found similar to protein sequences in the 2 model species Click here for file

Additional file 8 **List of shrimps' group 4 ESTs which matched to proteins in UniProt database**. This file lists 9,108 EST ID which did not find matching to proteins in the 2 model species but found matching to proteins from other species, with function and name of hit species. Click here for file
